# Correction: Alopecia areata patients show deficiency of FOXP_3_+CD_39_+ T regulatory cells and clonotypic restriction of Treg TCRβ-chain, which highlights the immunopathological aspect of the disease

**DOI:** 10.1371/journal.pone.0222473

**Published:** 2019-09-09

**Authors:** Fatma N. Hamed, Annika Åstrand, Marta Bertolini, Alfredo Rossi, Afsaneh Maleki-Dizaji, Andrew G. Messenger, Andrew J. G. McDonagh, Rachid Tazi-Ahnini

The following information is missing from the Funding section: MB received funding from the National Alopecia Areata Foundation (USA) and Associazione Mediterranea Alopecia Areata (Italy).
