# Supplementary material for: Alopecia areata patients show deficiency of FOXP3+CD39+ T regulatory cells and clonotypic restriction of Treg TCRβ-chain, which highlights the immunopathological aspect of the disease
Source: PLoS One. 2019 Jul 5;14(7):e0210308. doi: 10.1371/journal.pone.0210308 (PMC6611701; doi:10.1371/journal.pone.0210308)
Supplement: S1 Material and Methods — (DOCX) [file pone.0210308.s001.docx]

## DNA extraction for NGS

10^6^ PBMCs isolated from heparinized venous blood by density gradient purification as described in section 2.2.2, and 1x10^4^ CD4+CD25+FOXP3+ Tregs sorted by FACS technique underwent DNA extraction using two different kits. Genomic DNA (g-DNA) extraction from PBMCs was performed using Quick-gDNA extraction Miniprep (D-3006, Zymo research). Briefly, the lysis step was performed by adding 500μl of genomic lysis buffer to the cell pellet incubated 5-10mins at room temperature after mixing. The mixture was then transferred to a zymo-spin column in a collection tube and centrifuged at 10,000xg for one minute. After discarding the flow through, a DNA binding step was carried out by adding 200μl of DNA pre-wash buffer and centrifuging at the same speed for one minute, which was followed by washing using 500μl of g-DNA wash buffer. After the wash step, DNA elution was performed by adding 50μl elution buffer and incubated at RT for 2-5mins before centrifuging at top speed to elute the DNA. The eluted DNA was stored at -20ºC.

DNA extraction from FACs sorted Treg cells was performed using a kit that is suitable for paraffin fixed cells, Pico Pure DNA extraction kit (KIT0103, Applied Biosystems). 155μl of reconstitution buffer was added to one vial of proteinase K provided in the kit to construct the extraction solution. 150μl of extraction solution was added to the FACS sorted cell pellet and incubated at 65^◦^C for 12hrs, and then incubated at 95^◦^C for 10mins to deactivate the proteinase K and stored at -20^◦^C. DNA samples were then stored at -80^◦^C to be used for multiplex PCR reaction.

## Multiplex- PCR amplification of the TCRB CDR3 region

TCRβ CDR3 was defined according to International Immunogenetic collaboration (Yousfi Mondo et al., 2004): TCRβ CDR3 begins with the second conserved cysteine encoded by the 3′ position of the Vβ gene segment and ended with the conserved phenylalanine encoded by the 5′ position of the Jβ gene segment. Multiplex-PCR system was used to amplify the rearranged regions of TCRβ CDR3 from genomic DNA using 39 forward primers specific to TCR Vβ segments and 13 reverse primers each specific to a TCR Jβ segment (Primer sequences in appendix 1) to generate a template library for analyses using Genome Analyzer. 50μL PCR reaction was set at 0.8μM forward (F) primers pool (22nM for each unique TCR Vβ F primer), 1.0 μM reverse (R) primers pool (77nM for each unique TCR Jβ primer), 200ng g-DNA, 1× QIAGEN Multiplex PCR master mix and 5% Q solution (QIAGEN). The PCR reaction was performed under thermal cycling conditions listed in S1 Table. A clean PCR product using this protocol was obtained of about 500bp size as show in S1 Fig.

## Library preparation

All experiments in library preparation stage were performed by Mr Matt Wyles in the Sheffield Institute for Translational Neuroscience (SITraN) under guidance of Dr. Paul Heath, University of Sheffield.

#### DNA quantification

Prior to library construction, an accurate measurement of the concentration of PCR products was performed using Quibt ds DNAHS assay (Q32851, Invitrogen). The assay is simple involving adding diluted quibt working buffer provided in the kit to the standards and the samples in volumes stated in the protocol and incubated for 2mins at room temperature. The concentration of the samples was then calculated automatically using Qubit^®^ Fluorometer.

#### DNA Fragmentation

The size of DNA fragments (multiplex PCR product of PBMCs samples) was around 500bps, which cannot be sequenced in full by Hiscan SQ Illumina sequencer as it performs paired-end sequencing, coupled with 2× 100 bp read lengths. Therefore, DNA was first digested using the NEB double stranded DNA fragmentase enzyme (M03485, NEB) into appropriate size following the manufacturer’s protocol.

The digestion step was followed by an accurate determination of DNA fragments size using Agilent High sensitivity DNA assay (5067-4626, Agilent). The technique involves separating the samples based on their size by electrophoresis after loading them in Agilent chip. The travel time of samples through the micro-channels is based on their size where smaller fragments migrate faster. The chip was then read on an Aligent 2100 Bioanalyser involving two key metrics; fluorescent intensity units (FU) that depends on the sample concentration and migration time, which relates to the size of the protein. The Agilent2100 bioanalyser software generated graphs representing migration time in seconds (S) on X axis against the fluorescent units (FU) in the Y axis for the ladder (S2A Fig). The software then calculated the size of DNA fragments based on standard curve plotting the migration time against known ladder sizes, and the results depicted manually on each peak in bp (S2A Fig), which is then used to measure the size of DNA fragments for each well from the migration time. The samples (S2B Fig) were successfully fragmented.

#### DNA Purification

Bead purification of the PCR products was performed as described by supplier AgenCourt AMPure XP (A63881, Beckman Coulter) to remove excess enzymes, primers, salts and nucleotide. The Agencourt AMPure XP Purification systems utilize solid-phase paramagnetic bead technology for high-throughput purification of PCR amplicons where 1.8μl of AMPure XP buffer was added to 1.0μl of PCR product and incubated for 5mins at room temperature, the beads-containing buffer selectively bind to PCR amplicons. The plate was then placed onto the Agencourt magnetic rack for 2mins before aspirating the cleared supernatant. The bead-bound PCR product was then washed 2x with 200μl of 80% ethanol to remove contaminants and finally purified PCR was eluted from the beads by adding 40μl of elution buffer to each well and incubated for 2mins at room temperature after removing the plate from the magnet. The plate was then replaced onto the magnet for 1min before transferring the eluent (purified sample) into clean tubes to be used in final library preparation.

#### End library preparation

Sequencing libraries were generated using the standard protocol in the NEB Ultra II DNA library prep kit for Illumina (E7645S, NEB) summarised in S3 Figure. First, end repair of the fragmented DNA was performed by adding dA tail at 3’ end of the fragments and phosphorylation at 5’ end. In this reaction, 50μl of fragmented DNA added to 3μl of the NEBNext Ultra II End Prep Enzyme Mix and 7μl of NEBNext Ultra II End Prep reaction buffer and mixed very well. The reaction was run in the thermocycler with the following setup: 30 minutes at 20°C, 30 minutes at 65°C and hold at 4°C.

That was followed by adaptor ligation; platform-specific adapters serve as a template for the PCR reaction for final library preparation. Adaptor, which is self-complimentary oligonucleotides forming a stem-loop containing uracil, was added to the DNA fragments. The following buffers were added directly to end repair reaction; 30μl of NEBNext Ultra II Ligation Master Mix, 1μl of NEBNext Ligation Enhancer and 2.5μl NEBNext Adaptor for Illumina, mixed 10 times and incubated for 15mins at 20^◦^C in the thermocylcer. That was followed by adding 3μl of USER™ (Uracil-Specific Excision Reagent) enzyme to the ligation mixture and incubated at 37ºC for 15mins in order to generate a single nucleotide gap in the adaptor at the uracil residue to open the loops.

Finally, clean-up was performed to remove excess enzymes using Agencourt AMPure XP beads as described previously and a high sensitivity DNA bioanalyser chip (Agilent) was performed to assess the size of the DNA fragments and the amount of the final library was quantified using the Qubit kit.

#### Library amplification

The adaptor-ligated DNA was amplified by PCR reaction (S2 Table) where barcoding sequence identifying each sample was added to the DNA fragments and indexed by forward and reverse primers for paired-end sequencing (P5 and P7 Primer) adding termini that bind to the oligonucleotides on the flow cell surface.

*P5 PCR Primer:*

5’ AATGATACGGCGACCACCGAGATCTACACTCTTTCCCTACACGA 3’

*P7 PCR Primer:*

5’ CAAGCAGAAGACGGCATACGAGAT 3’.

All samples were indexed uniquely so they could be pooled together and sequenced in one lane of a HiScan SQ using a 2x100bp high output run. The reaction was run in the thermocycler using thermal profile in S3 Table.

A final clean-up using beads as described before was performed and the library size was about 300bp. The total DNA concentration in the final library (S4 Table) was calculated by Quibt.

## Quality control

The quality of the NGS data can be affected by a series of steps involved the sequencing process such as library preparation, base calling and read alignment. Therefore, several quality metrics were applied to assess the effect of each step of the upstream workflow on the data output. The sequence analysis viewer of Illumina provides quality control charts during the run to monitor the run quality and reliability. The base calling accuracy is one of the main quality metrics; it refers to the accuracy of the sequencer to recall the right base. This is measured by the Phred quality score (Q score), which calculates the probability of calling the incorrect base using the formula (Q=-10 log_10_ P) where P is the base calling error probability giving figures as shown in table 1.1 where the higher Q score means the higher probability of calling the right base during the sequencing process. In this NGS experiment, 89% of the data was above Q30 indicating the probability of calling the right base was 99.9% (S5 Fig).

The distribution of quality metric throughout the 200 cycles run was calculated by Illumina software. The clusters became unstable over the length of a 200-cycle run as it took 11 days. Therefore, it was observed that the quality was high initially, and dropped off gradually, however, it remained above Q30. Note the decline in quality at cycle 50 and 150 and indexing step at about 100 cycle (S5 Fig).

The drop in the quality at cycles 50, 100 and 150 can be explained by looking at signal intensity (S6 Fig). This shows the signal intensity for each base (A, C, G, T) added to each cluster and imaged over the course of the entire 200 sequencing cycles. Cycles 50 and 150 show a spike in intensity where the laser receives a power boost. Finally, the change in intensity during the middle part of the run is due to the index read where the barcodes added during the library preparation were sequenced during that time.

Cluster density is another key metric that impacts sequencing performance and the quality of the run, while under-clustering causes lower data output, over-clustering lowers the Q score due to introducing sequencing artefact ([https://support.illumina.com/content/ 770-2014-038.pdf](https://support.illumina.com/content/dam/illumina-marketing/documents/products/other/miseq-overclustering-primer-770-2014-038.pdf)). Illumina has a so-called Chastity filter to measure the purity of the signal and reads failing to pass the filter are considered less reliable and removed from the analysis. Therefore, the percentage of reads pass-filter (%PF) is an important quality indicator and over-clustering negatively impacts on the %PF causing its reduction. The study data in lane 1 had cluster density (blue box) of about 700,000 clusters per mm^2^, and they were overlapping with %PF quality filter (red box) indicating optimal generation of sequence data (S7 Fig).

## Primary analysis of TCR clonotype

The bioinformatics analysis was performed in collaboration with Dr. Afsaneh Maleki-Dizaji, a research fellow in computational biology, Department of Computer Science, University of Sheffield. Reads of the DNA sequence were paired endwise. The Illumina sequencer generated bcl files, which were converted to fastq files by the bcl2fastq program. An average of 7.7 million total raw reads were obtained from each sample, and 7 million reads that met the quality requirements after removal of low-quality reads were aligned to the TCRβ CDR3 human genome using the MIXCR pipeline (S6 and S7 Tables).

**Appendix 1**

Multiplex PCR primer sequences for V and J segments.

| **TRBV gene segment(s)** | **Primer sequence** |
| --- | --- |
| **TRBV2** | TCAAATTTCACTCTGAAGATCCGGTCCACAA |
| **TRBV3-1** | GCTCACTTAAATCTTCACATCAATTCCCTGG |
| **TRBV4-1** | CTTAAACCTTCACCTACACGCCCTGC |
| **TRBV(4-2, 4-3)** | CTTATTCCTTCACCTACACACCCTGC |
| **TRBV5-1** | GCTCTGAGATGAATGTGAGCACCTTG |
| **TRBV(5-4, 5-5, 5-6, 5-7, 5-8)** | GCTCTGAGCTGAATGTGAACGCCTTG |
| **TRBV(6-2, 6-3)** | GCTGGGGTTGGAGTCGGCTG |
| **TRBV6-4** | CCCTCACGTTGGCGTCTGCTG |
| **TRBV6-8** | CACTCAGGCTGGTGTCGGCTG |
| **TRBV6-9** | CGCTCAGGCTGGAGTCAGCTG |
| **TRBV7-2** | CACTCTGACGATCCAGCGCACAC |
| **TRBV7-3** | CTCTACTCTGAAGATCCAGCGCACAG |
| **TRBV7-4** | CCACTCTGAAGATCCAGCGCACAG |
| **TRBV7-7** | CCACTCTGACGATTCAGCGCACAG |
| **TRBV7-9** | CACCTTGGAGATCCAGCGCACAG |
| **TRBV9** | GCACTCTGAACTAAACCTGAGCTCTCTG |
| **TRBV10-1** | CCCCTCACTCTGGAGTCTGCTG |
| **TRBV10-2** | CCCCCTCACTCTGGAGTCAGCTA |
| **TRBV10-3** | CCTCCTCACTCTGGAGTCCGCTA |
| **TRBV(11-1,11-2, 11-3)** | GAGGCTCAAAGGAGTAGACTCCACTCT |
| **TRBV(11-1, 11-3)** | CCACTCTCAAGATCCAGCCTGCAG |
| **TRBV(12-3, 12-4, 12-5)** | CCACTCTGAAGATCCAGCCCTCAG |
| **TRBV13** | CATTCTGAACTGAACATGAGCTCCTTGG |
| **TRBV14** | CTACTCTGAAGGTGCAGCCTGCAG |
| **TRBV15** | GATAACTTCCAATCCAGGAGGCCGAACA |
| **TRBV16** | CTGTAGCCTTGAGATCCAGGCTACGA |
| **TRBV18** | GCATCCTGAGGATCCAGCAGGTAG |
| **TRBV19** | CCTCTCACTGTGACATCGGCCC |
| **TRBV20-1** | CTTGTCCACTCTGACAGTGACCAGTG |
| **TRBV24-1** | CTCCCTGTCCCTAGAGTCTGCCAT |
| **TRBV25-1** | CCCTGACCCTGGAGTCTGCCA |
| **TRBV27** | CCCTGATCCTGGAGTCGCCCA |
| **TRBV28** | CTCCCTGATTCTGGAGTCCGCCA |
| **TRBV29-1** | CTAACATTCTCAACTCTGACTGTGAGCAACA |
| **TRBV30** | CGGCAGTTCATCCTGAGTTCTAAGAAGC |

| TRBJ gene segment Primer sequence | |
| --- | --- |
| TRBJ1-1 | TTACCTACAACTGTGAGTCTGGTGCCTTGTCCAAA |
| TRBJ1-2 | ACCTACAACGGTTAACCTGGTCCCCGAACCGAA |
| TRBJ1-3 | ACCTACAACAGTGAGCCAACTTCCCTCTCCAAA |
| TRBJ1-4 | CCAAGACAGAGAGCTGGGTTCCACTGCCAAA |
| TRBJ1-5 | CTTACCTAGGATGGAGAGTCGAGTC |
| TRBJ1-6 | CTGTCACAGTGAGCCTGGTCCCATTCCC |
| TRBJ2-1 | CGGTGAGCCGTGTCCCTGGCCCGAA |
| TRBJ2-2 | CCAGTACGGTCAGCCTAGAGCCTTCTCCAAA |
| TRBJ2-3 | ACTGTCAGCCGGGTGCCTGGGCCAAA |
| TRBJ2-4 | AGAGCCGGGTCCCGGCGCCGAA |
| TRBJ2-5 | GGAGCCGCGTGCCTGGCCCGAA |
| TRBJ2-6 | GTCAGCCTGCTGCCGGCCCCGAA |
| TRBJ2-7 | GTGAGCCTGGTGCCCGGCCCGAA |
