# Supplementary material for: Alopecia areata patients show deficiency of FOXP3+CD39+ T regulatory cells and clonotypic restriction of Treg TCRβ-chain, which highlights the immunopathological aspect of the disease
Source: PLoS One. 2019 Jul 5;14(7):e0210308. doi: 10.1371/journal.pone.0210308 (PMC6611701; doi:10.1371/journal.pone.0210308)
Supplement: S1 Table — (DOCX) [file pone.0210308.s002.docx]

| **Reaction** | **Duration/Temperature** | **Cycle** |
| --- | --- | --- |
| Hot start activation of polymerase | 15mins at 95◦C | 1 |
| Denaturation | 30sec at 94◦C | 40 |
| Annealing | 90sec at 59◦C |  |
| Extension | 1min at 72ᵒC |  |
| Final extension | 10mins at 72◦C | 1 |
