# Supplementary material for: Alopecia areata patients show deficiency of FOXP3+CD39+ T regulatory cells and clonotypic restriction of Treg TCRβ-chain, which highlights the immunopathological aspect of the disease
Source: PLoS One. 2019 Jul 5;14(7):e0210308. doi: 10.1371/journal.pone.0210308 (PMC6611701; doi:10.1371/journal.pone.0210308)
Supplement: S2 Table — (DOCX) [file pone.0210308.s003.docx]

| **Reagent** | **Volume** |
| --- | --- |
| Adaptor-ligated DNA fragments | 15 μl |
| NEBNext Ultra II Q5 Master Mix | 25 μl |
| Index Primer/i7 Primer | 5 μl |
| i5 Primer | 5 μl |
| Total volume | 50 μl |
