# Supplementary material for: Alopecia areata patients show deficiency of FOXP3+CD39+ T regulatory cells and clonotypic restriction of Treg TCRβ-chain, which highlights the immunopathological aspect of the disease
Source: PLoS One. 2019 Jul 5;14(7):e0210308. doi: 10.1371/journal.pone.0210308 (PMC6611701; doi:10.1371/journal.pone.0210308)
Supplement: S3 Table — (DOCX) [file pone.0210308.s004.docx]

| **Reaction** | **Duration/Temperature** | **Cycle** |
| --- | --- | --- |
| Initial Denaturation | 30s at 98◦C | 1 |
| Denaturation | 10s at 98◦C | 8 |
| Annealing and elongation | 75s at 65◦C |  |
| Final extension | 5mins at 65◦C | 1 |
