# Supplementary material for: Alopecia areata patients show deficiency of FOXP3+CD39+ T regulatory cells and clonotypic restriction of Treg TCRβ-chain, which highlights the immunopathological aspect of the disease
Source: PLoS One. 2019 Jul 5;14(7):e0210308. doi: 10.1371/journal.pone.0210308 (PMC6611701; doi:10.1371/journal.pone.0210308)
Supplement: S4 Table — (DOCX) [file pone.0210308.s005.docx]

| **Sample ID** | **Fragment size** | **Qubit** | **nM** |
| --- | --- | --- | --- |
| AA29 | 326 | 33 | 155.7338 |
| AA28 | 326 | 22.4 | 105.7102 |
| AA27 | 326 | 15 | 70.78811 |
| AA26 | 326 | 17.2 | 81.17036 |
| AA24 | 326 | 34.6 | 163.2846 |
| AA22 | 326 | 14.8 | 69.84427 |
| AA21 | 326 | 41.8 | 197.2629 |
| AA13 | 326 | 11.2 | 52.85512 |
| AA10 | 326 | 10.7 | 50.49552 |
| AA7 | 326 | 19.8 | 93.4403 |
| HC18 | 326 | 8.45 | 39.8773 |
| HC17 | 326 | 17.7 | 83.52997 |
| HC16 | 326 | 44.9 | 211.8924 |
| HC15 | 326 | 26.9 | 126.9467 |
| HC13 | 326 | 11.5 | 54.27088 |
| HC03 | 326 | 15.5 | 73.14771 |
| HC01 | 326 | 26.2 | 123.6432 |
| AA34 | 326 | 72.9 | 344.0302 |
| AA33 | 326 | 43.8 | 206.7013 |
| AA32 | 326 | 72.7 | 343.0864 |
| HC16 | 326 | 8.51 | 40.16045 |
| HC15 | 326 | 21.3 | 100.5191 |
| AA26 | 326 | 14.4 | 67.95658 |
| HC01 | 326 | 10.1 | 47.66399 |
