# Supplementary material for: Alopecia areata patients show deficiency of FOXP3+CD39+ T regulatory cells and clonotypic restriction of Treg TCRβ-chain, which highlights the immunopathological aspect of the disease
Source: PLoS One. 2019 Jul 5;14(7):e0210308. doi: 10.1371/journal.pone.0210308 (PMC6611701; doi:10.1371/journal.pone.0210308)
Supplement: S5 Table — (DOCX) [file pone.0210308.s006.docx]

| Phred Q Score | Probability of incorrect base call | Accuracy of Base call |
| --- | --- | --- |
| 10 | 1 in 10 | 90% |
| 20 | 1 in 100 | 99% |
| 30 | 1 in 1000 | 99.9% |
| 40 | 1 in 10,000 | 99.99% |
| 50 | 1 in 100,000 | 99.999% |
