# Supplementary material for: Alopecia areata patients show deficiency of FOXP3+CD39+ T regulatory cells and clonotypic restriction of Treg TCRβ-chain, which highlights the immunopathological aspect of the disease
Source: PLoS One. 2019 Jul 5;14(7):e0210308. doi: 10.1371/journal.pone.0210308 (PMC6611701; doi:10.1371/journal.pone.0210308)
Supplement: S6 Table — F = FACS (Treg sorted by FACs), P = PBMC, HC = Healthy control, AA = alopecia areata patients. (DOCX) [file pone.0210308.s007.docx]

| **Sample ID** | **Clinical subtype** | **Number of T cells**  **PBMCs 10^6^** | **Number of T reg** | **DNA concentration nM** | **Total sequence read** | **% of assembled reads** |
| --- | --- | --- | --- | --- | --- | --- |
| FAA26 | AU | 10 | 52326 | 17 | 6362770 | 95% |
| FAA32 | AA | 22 | 17746 | 73 | 7291450 | 77% |
| FAA33 | AA | 11 | 2944 | 44 | 9373820 | 71% |
| FAA34 | AU | 28 | 11531 | 73 | 3731882 | 84% |
| FHC01 | NA | 36 | 13000 | 26 | 3799472 | 93% |
| FHC15 | NA | 15 | 43454 | 21 | 4825830 | 77% |
| FHC16 | NA | 17 | 19000 | 9 | 2978560 | 94% |
| FHC01 | NA | 36 | 13000 | 10 | 2625519 | 98% |

AA: patchy alopecia areata; AT: alopecia totalis; AU: alopecia universalis. NA: not applicable
