# Supplementary material for: Alopecia areata patients show deficiency of FOXP3+CD39+ T regulatory cells and clonotypic restriction of Treg TCRβ-chain, which highlights the immunopathological aspect of the disease
Source: PLoS One. 2019 Jul 5;14(7):e0210308. doi: 10.1371/journal.pone.0210308 (PMC6611701; doi:10.1371/journal.pone.0210308)
Supplement: S7 Table — P = PBMC, HC = Healthy control, AA = alopecia areata patients. (DOCX) [file pone.0210308.s008.docx]

| **Sample ID** | **Clinical type** | **DNA concentration nM** | **Total sequence read** | **% of assembled reads** |
| --- | --- | --- | --- | --- |
| PAA7 | AA | 20 | 8919572 | 97% |
| PAA10 | AU | 11 | 13198354 | 96% |
| PAA13 | AA | 11 | 17074228 | 97% |
| PAA21 | AT | 42 | 7932050 | 97% |
| PAA22 | AU | 15 | 11000178 | 97% |
| PAA24 | AU | 35 | 5477294 | 97% |
| PAA26 | AU | 17 | 9062926 | 97% |
| PAA27 | AA | 71 | 5839794 | 97% |
| PAA28 | AT | 105 | 8076702 | 97% |
| PAA29 | AA | 155 | 5721290 | 97% |
| PHC01 | NA | 26 | 2625519 | 98% |
| PHC03 | NA | 16 | 13097328 | 97% |
| PHC13 | NA | 12 | 8083120 | 97% |
| PHC15 | NA | 27 | 5530536 | 97% |
| PHC16 | NA | 45 | 3784520 | 97% |
| PHC17 | NA | 18 | 6588222 | 98% |
| PHC18 | NA | 8 | 24908242 | 97% |

AA: patchy alopecia areata; AT: alopecia totalis; AU: alopecia universalis. NA: not applicable
