# Supplementary figures and images for: Alopecia areata patients show deficiency of FOXP3+CD39+ T regulatory cells and clonotypic restriction of Treg TCRβ-chain, which highlights the immunopathological aspect of the disease
Source: PLoS One. 2019 Jul 5;14(7):e0210308. doi: 10.1371/journal.pone.0210308 (PMC6611701; doi:10.1371/journal.pone.0210308)

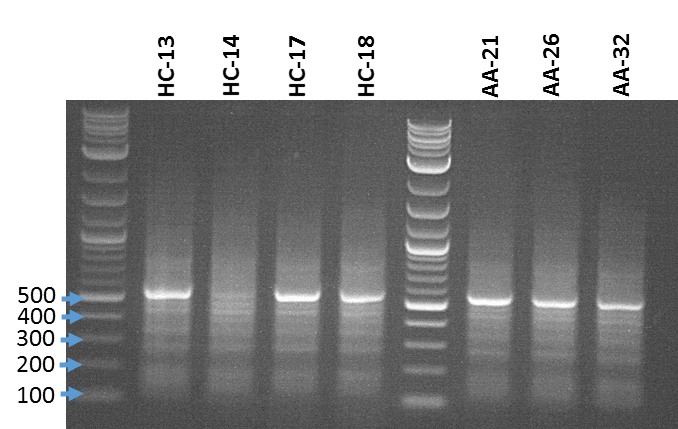

Supplement: S1 Fig — Bands are of about 500bps size. (TIF) [file pone.0210308.s009.tif]

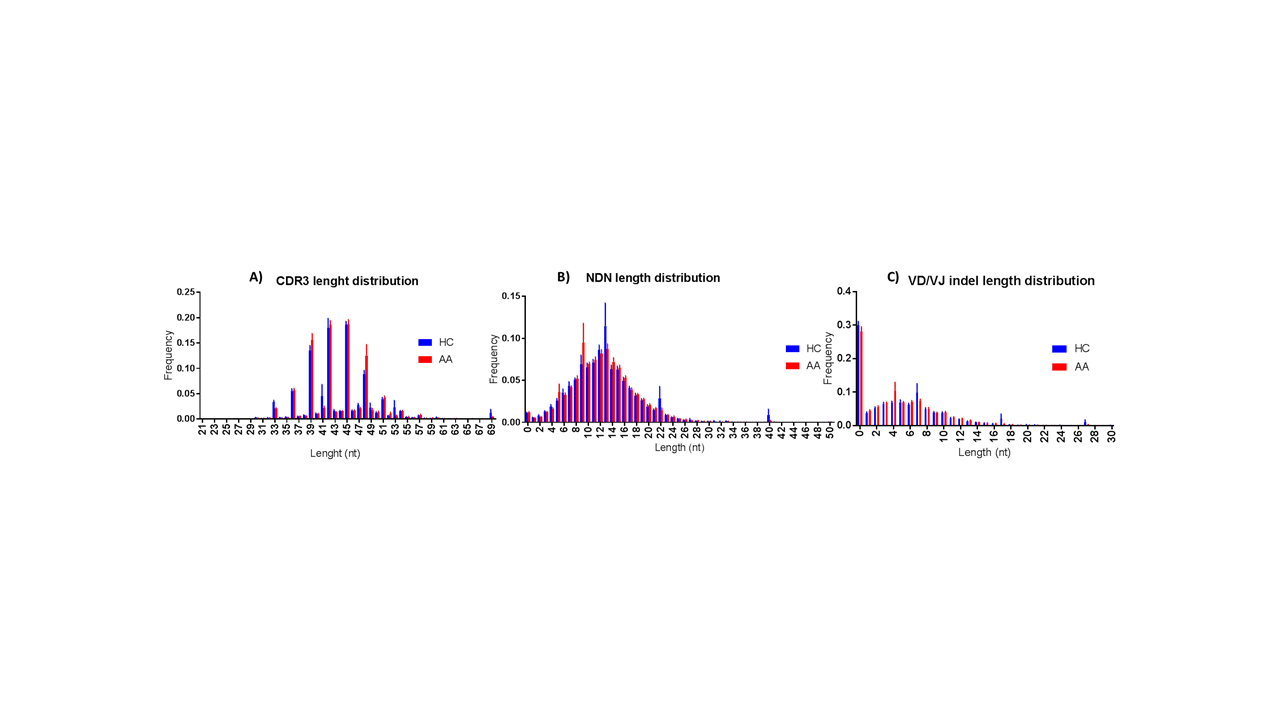

Supplement: S2 Fig — A. Plot shows the migration time (seconds) against fluorescent intensity (FU) of the ladder with known size. B. Representative electropherogram of the DNA library after digestion where the peak point is at about 300bp. (TIF) [file pone.0210308.s010.tif]

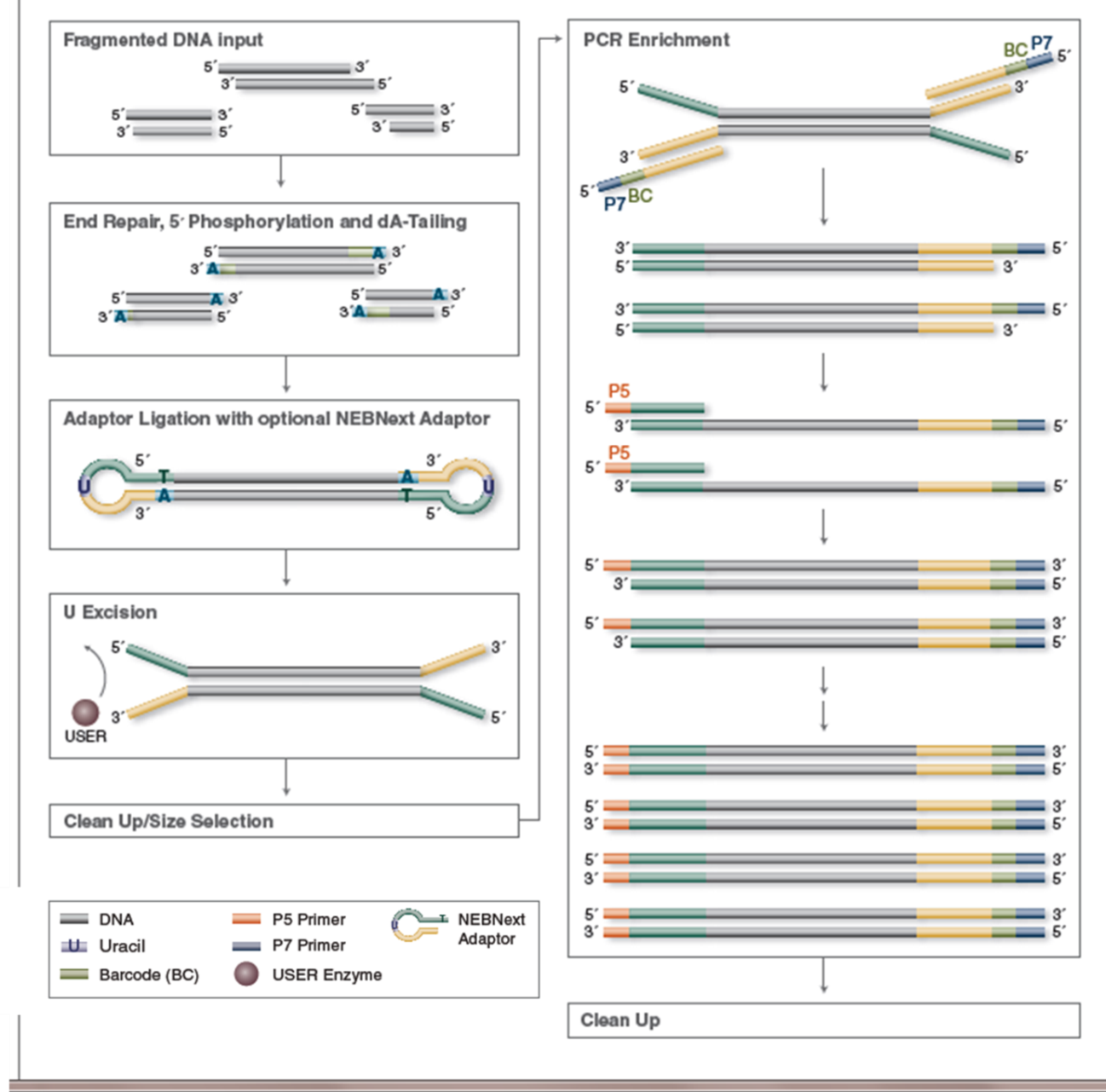

Supplement: S3 Fig — First, DNA fragments undergo end repair of the fragmented DNA by adding dA tail at 3’ end of the fragments and phosphorylation at 5’ end. Secondly, adapter ligation is performed. Thirdly, the adapter loop is cut by USER enzyme, and the library undergoes clean up step. Finally, PCR amplification is performed using P5 and P7 indexed primers and involves inserting barcode for each sample. (TIF) [file pone.0210308.s011.tif]

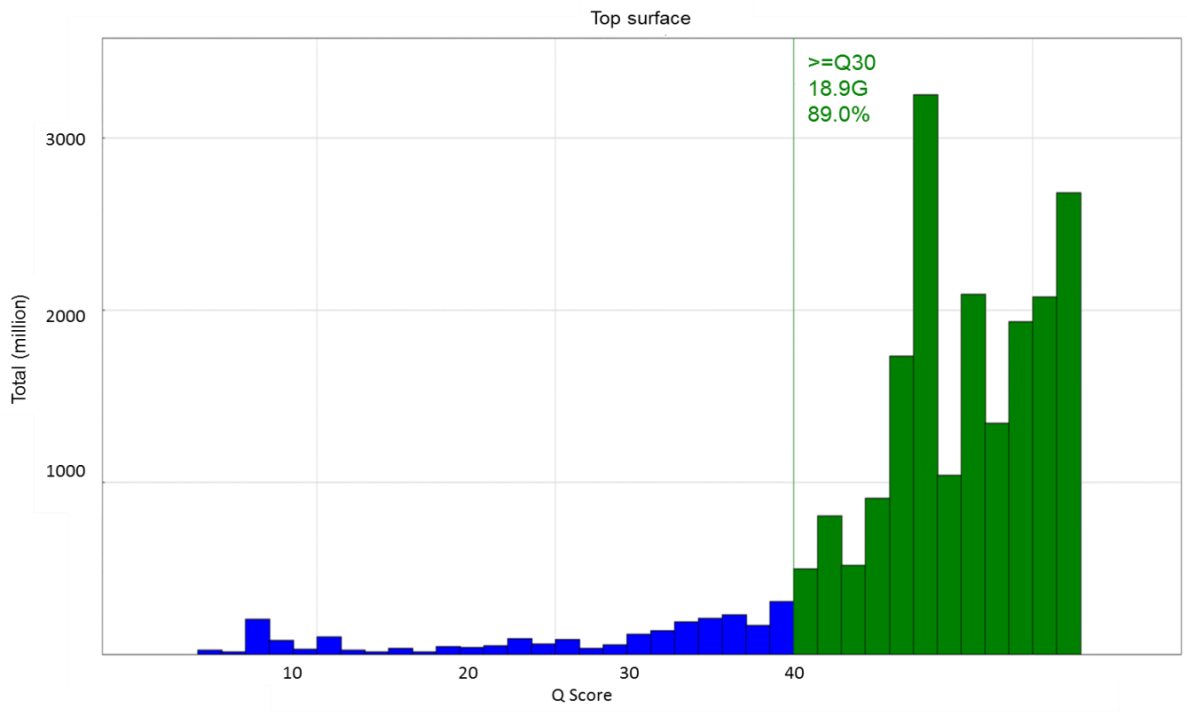

Supplement: S4 Fig — The number of sequencing cycles on X-axis and the signal intensity on Y-axis. Bases were visualized in different colors, A = red, C = green, G = Blue and T = black. (TIF) [file pone.0210308.s012.tif]

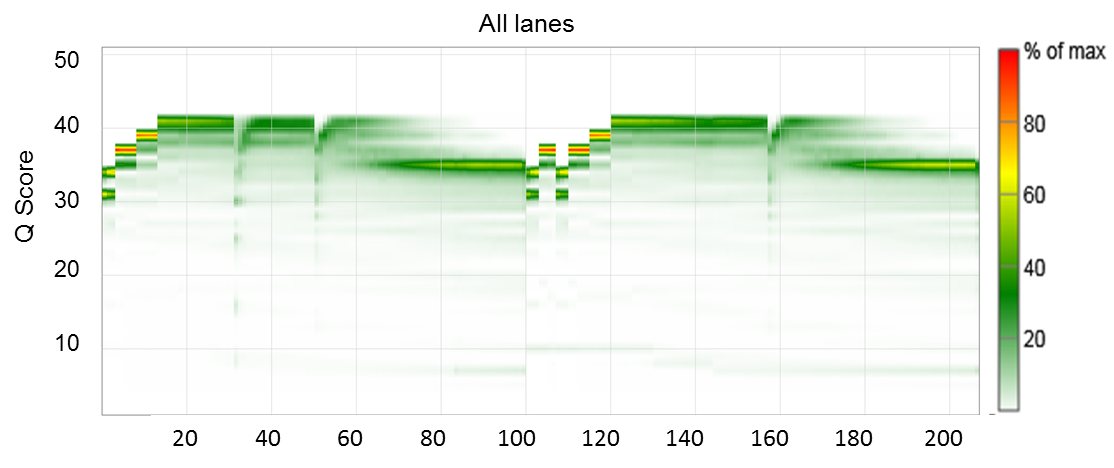

Supplement: S5 Fig — Q score was above 30 thought the major time of the run, drops at about 50, 100 and 150 can be seen. (TIF) [file pone.0210308.s013.tif]

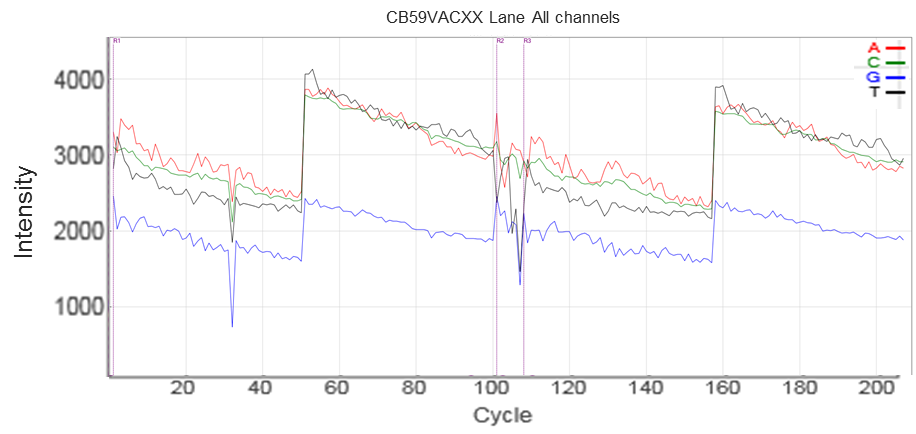

Supplement: S6 Fig — The number of sequencing cycles on X-axis and the signal intensity on Y-axis. Bases were visualised in different colors, A = red, C = green, G = green and T = black. (PNG) [file pone.0210308.s014.png]

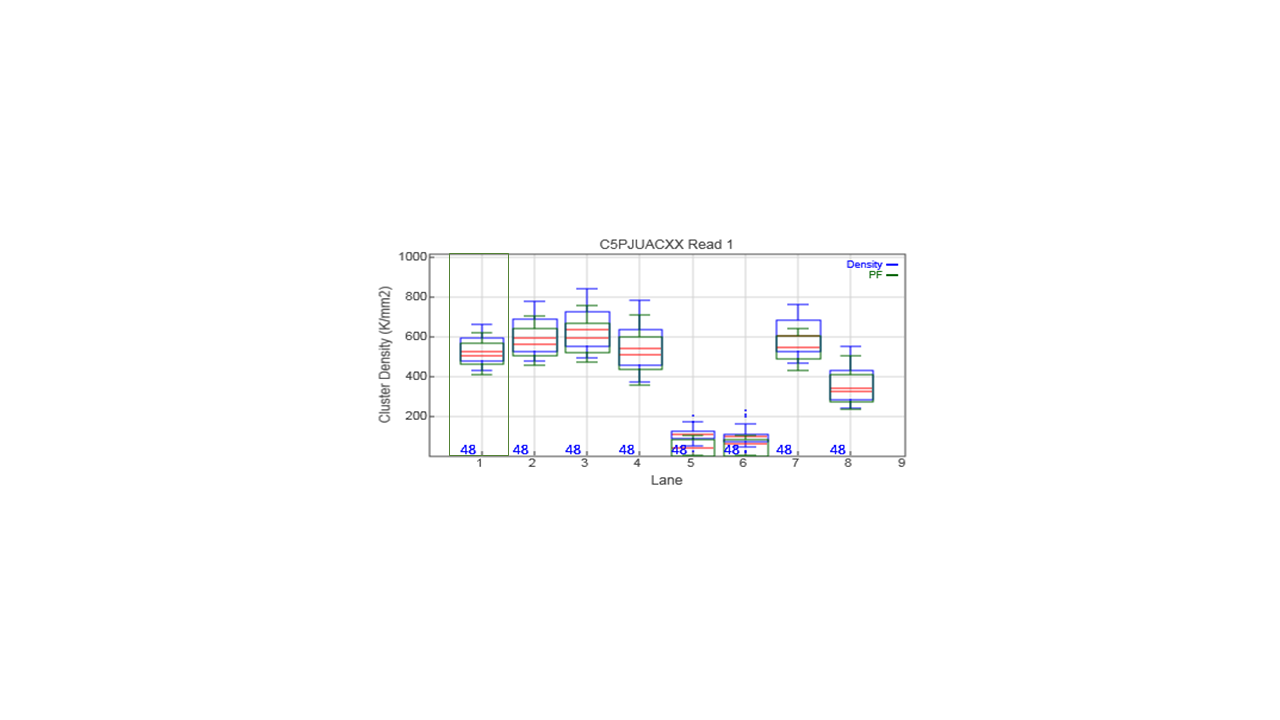

Supplement: S7 Fig — The lanes (1–8) in flow cell are represented on the X-axis. The cluster density is represented on the Y-axis. The samples were loaded on lane 1 (23 samples). Cluster density in the blue box and %PF in the green box and the median shown by a red line. The cluster density and %PF boxes are overlapping. (TIF) [file pone.0210308.s015.tif]
